# Novel Indole-Based Hydrazones as Potent Inhibitors of the α-class Carbonic Anhydrase from Pathogenic Bacterium *Vibrio cholerae*

**DOI:** 10.3390/ijms21093131

**Published:** 2020-04-29

**Authors:** Kübra Demir-Yazıcı, Özlen Güzel-Akdemir, Andrea Angeli, Claudiu T. Supuran, Atilla Akdemir

**Affiliations:** 1 Department of Pharmaceutical Chemistry, Faculty of Pharmacy, Istanbul University, Beyazit, 34116 Istanbul, Turkey; 2 Neurofarba Department, Section of Pharmaceutical and Nutriceutical Sciences, Università degli Studi di Firenze, Via U. Schiff 6, 50019 Sesto Fiorentino (Florence), Italy; 3 Centre of Advanced Research in Bionanoconjugates and Biopolymers Department, “Petru Poni” Institute of Macromolecular Chemistry, 700487 Iasi, Romania; 4 Computer-aided Drug Discovery Laboratory, Department of Pharmacology, Faculty of Pharmacy, Bezmialem Vakif University, Fatih, 34093 Istanbul, Turkey

**Keywords:** carbonic anhydrase, indole, sulfonamides, hydrazones, *Vibrio cholerae*, enzyme inhibition, molecular modeling

## Abstract

Due to the increasing resistance of currently used antimicrobial drugs, there is an urgent problem for the treatment of cholera disease, selective inhibition of the α-class carbonic anhydrases (CA, EC 4.2.1.1) from the pathogenic bacterium *Vibrio cholerae* (VcCA) presents an alternative therapeutic target. In this study, a series of hydrazone derivatives, carrying the 2-(hydrazinocarbonyl)-3-phenyl-1*H*-indole-5-sulfonamide scaffold, have been evaluated as inhibitors of the VcCA with molecular modeling studies. The results suggest that these compounds may bind to the active site of VcCA. To verify this, VcCA enzyme inhibition studies were performed and as predicted most of the tested compounds displayed potent inhibitory activities against VcCA with three compounds showing *K*_I_ values lower than 30 nM. In addition, all these compounds showed selectivity for VcCA and the off-targets hCA I and II.

## 1. Introduction

Cholera is an acute diarrheal infection caused by pathogenic Gram-negative comma-shaped bacterium *Vibrio cholerae* and spreads by the ingestion of contaminated water or food. *Vibrio cholerae* is divided into 200 serogroups based on O antigen, but only the O1 or O139 serogroups are responsible for epidemic or pandemic cholera [1,2,3]. This highly virulent bacteria causes characteristic rice-water stool, typical clinical symptoms like dehydration, hypovolemic shock, acidosis, and if not well-treated leads to death [4,5]. Every year, there are roughly 1.3–4.0 million cases, and 21,000–143,000 deaths worldwide due to cholera and the last estimated global burden of cholera was approximately 1.2 million cases and 5654 deaths in 34 endemic countries, reported in 2017 by the World Health Organization (WHO) [6,7]. Asia has been cited as the starting point for seven cholera epidemics that have spread to most of Africa, Europe, and America since 1817 [8]. Between April 2017 and 2018, Yemen faced the world’s largest cholera outbreak, with 1,090,280 cases and 2275 deaths [9]. Cholera continues to be a global public health problem that often affects the nations with lower economic status and indicates the inequality and lack of social development between the countries [10,11]. 

Today, cholera is a preventable disease that can be eliminated by whole-cell oral cholera vaccines (OCV). There are three WHO-prequalified OCVs to date: Dukoral (Valneva, Sweden), Shanchol (Sanofi/Shanta Biotechnics, India), and Euvichol (Eubiologics, South Korea), which can be used in endemic cholera, situations with a high risk of cholera and cholera outbreaks [12,13]. For cholera treatment, the first step is stopping dehydration and the correction of ongoing electrolyte loss through fluid replacement with intravenous fluids like Ringer’s lactate and then oral rehydration solutions (ORS), which is readily prepared sugar and salt solution [14]. Antimicrobial agents can also be used as adjunct therapy to hydration and help to reduce the bacterial shedding and volume of diarrhea [15]. Antibiotics such as tetracycline, doxycycline, ciprofloxacin are the most common agents in therapy. In addition, macrolides like erythromycin or azithromycin present an option for children and pregnant women [16]. Considering the fact that the resistance of the current antimicrobial agents for cholera treatment is a growing problem hard to overcome, the development of new strategies and drugs with different mechanisms of action become an important approach to fight this mortal disease [17,18,19].

As an alternative drug target, three main classes of carbonic anhydrase enzymes (CA) from *Vibrio cholerae*, α, β, and γ were investigated for their potential for new antimicrobial drugs [20,21,22,23,24,25,26,27,28,29]. α-CA from *Vibrio cholerae* (VcCA), like all other carbonic anhydrase families (α-, β-, γ-, δ-, ζ-, η-, θ-, and ι-CAs), catalyzes the reversible conversion of carbon dioxide to a bicarbonate ion and a proton, and play an important role for the homeostasis and the virulence of *Vibrio cholerae* [30,31,32]. This Gram-negative bacterium colonizes the upper small intestine, where the amount of the sodium bicarbonate is at a high concentration. Bicarbonate is a potential inducer of virulence gene expression. If there are not enough transporter proteins, *V. cholerae* can increase cytosolic bicarbonate levels through the action of VcCA. Therefore, survival and virulence of this pathogen seems to be related to the utilization of the CA system [22,33]. Thus, inhibition of VcCA with selective carbonic anhydrase inhibitors offers a new aspect for cholera treatment. In our previous work, a new series of 2-(hydrazinocarbonyl)-3-phenyl-1*H*-indole-5-sulfonamide based hydrazones were synthesized and tested for their inhibitory profiles against tumor-related human carbonic anhydrase isozymes hCA IX/XII and against widely distributed off-targeted isoforms hCA I/II. In enzyme inhibition assays, these new molecules showed good inhibitory activity in the low nanomolar range and expressed selectivity over hCA I/II [34]. In this study, we first suggest that these compounds are potential inhibitors of VcCA with docking and molecular dynamics simulations. Subsequently, VcCA enzyme inhibition assays were performed to verify these compounds as novel inhibitors of VcCA, with selectivity over the widely distributed hCA I and II enzymes. These compounds may be used as leads in the search of new potent antimicrobial compounds for cholera treatment.

## 2. Results

### 2.1. VcCA Homology Model Construction and Molecular Dynamics Simulation

No crystal structure exists of the *Vibrio cholerae* α-CA enzyme (VcCA; Uniprot: A0A0X1L2C8). Therefore, a homology model was constructed. To this end, a BLAST search for protein crystal structures with similar sequences as the VcCA sequence was performed. This identified the α-CA from *Photobacterium profundum* (PpCA; pdb: 5hpj; 1.5 Å) as a template for the construction of VcCA homology models (query cover: 90%; identity: 59.45%).

**Figure 1:**
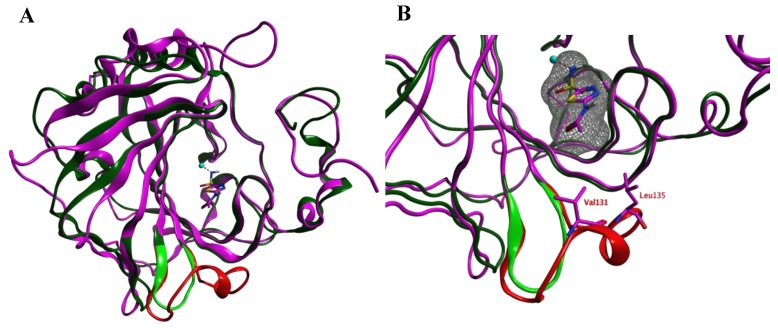
(**A**) The overlay of the hCA IX (purple and red) and PpCA (dark and light green) structures. The carbonic anhydrase enzymes (CA) inhibitor acetazolamide (purple) that is cocrystallized in the hCA IX structure is shown. (**B**) A zoom-in of the active sites of both enzymes reveals that the hCA IX loop (Ser124–Gly140; red) is larger compared to the PcCA loop (green) and approaches the cocrystallized ligand. The ligand surface is indicated in a grey mesh.

Prior to the homology modeling, the PpCA structure was superposed upon the hCA IX structure in complex with acetazolamide (azm) (3iai) to obtain insight into the differences between the two αCAs. These two structures show 53.6% sequence identity. Both structures share a reasonable structural similarity overall (RMSD: 2.221 Å; Cα-atoms; 211 residues) and between the active sites overall (RMSD: 0.402 Å; Cα-atoms; 13 residues). The loop between Ser124–Gly140 (hCA IX numbering), which is lining the active site and is located within 4.0 Å from azm, is much shorter and differently folded in the PpCA structure (Figure 1). 

**Figure 2:**
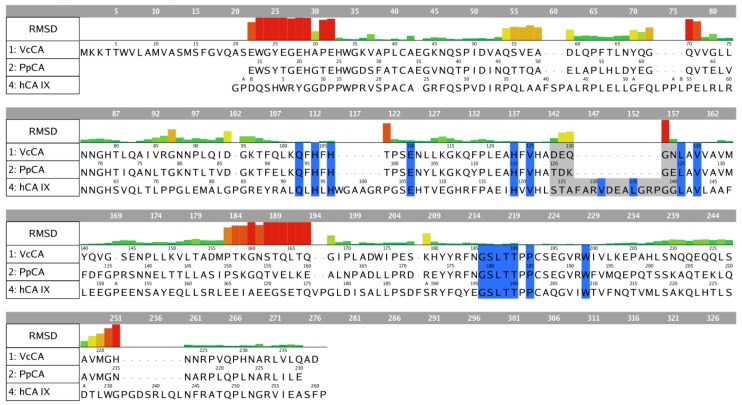
Sequence alignment of VcCA (UniProt: A0A0X1L2C8), PpCA (pdb: 5hpj) and hCA IX (pdb: 3iai). The residues indicated in blue belong to the binding pocket (within 4.5 Å of azm). The residues indicated in grey are part of the different folded loop near the pocket (Ser124–Gly140; hCA IX numbering). The RMSD per residue between PpCA and hCA IX is shown.

The RMSD values of the pocket residues of both enzymes (defined as all amino acids within 4.5 Å distance to azm, except for Val131 and Leu135) is rather low (0.404 Å over 14 residues; Figure 2). Visual inspection furthermore revealed that the active site zinc ion, the three zinc-binding His residues (His94, 96, and 119; hCA IX numbering), and the residues that are involved in the binding of azm (Leu198, Thr199, Thr200; hCA IX numbering) superpose well. Interestingly, these pocket amino acids are also conserved within the VcCA sequence (Figure 2). Therefore, the azm coordinates were copied into the PpCA structure. 

A total of 100 homology models were constructed of VcCA using the PpCA crystal structure as a template that also contained the azm coordinates from the hCA IX structure. The VcCA homology model with the lowest contact energy was selected for further optimization after the binding pose of azm was confirmed to be identical to the PpCa-azm template. Subsequently, all backbone atoms, all atoms of His104, 106, and 123 (counterparts of hCA IX His94, 96, and 119), and azm atoms were restrained and the VcCA-azm model was minimized (AMBER14:EHT). This minimization was repeated with a controlled release of restrains that allowed the restrained atoms to move further away from their original coordinates (standard deviation tether of 0.5, 1.0, and 1.5 Å, respectively). Finally, an energy minimization without restraints was performed to yield the final VcCA-azm model that was used for docking and molecular dynamics simulations.

**Figure 3:**
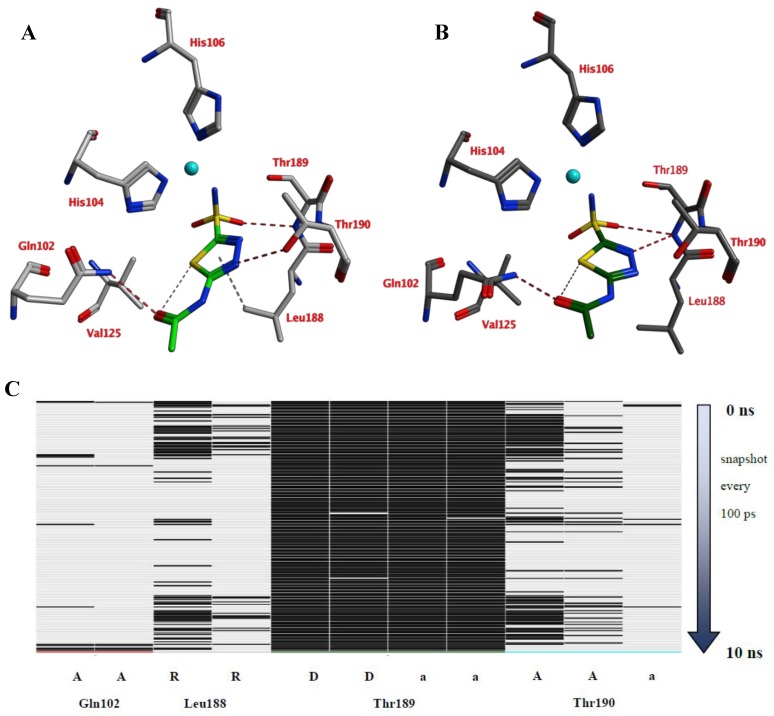

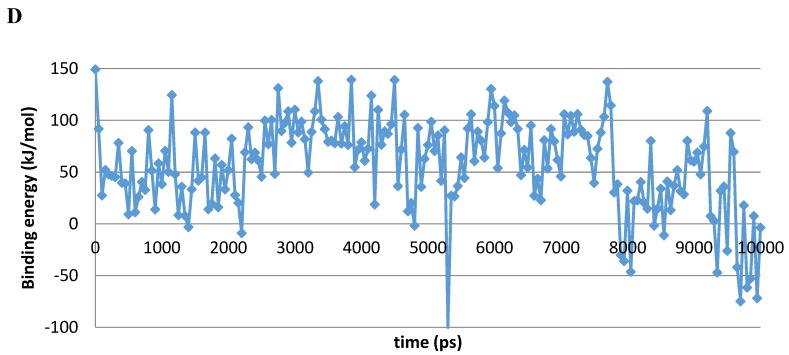
(**A**) The VcCA-azm model (t = 0 ns). (**B**) The VcCA-azm model after a 10 ns MD simulation. (**C**) The protein–ligand interaction fingerprint (PLIF) showing the interaction of azm with the VcCA binding pocket residues during the simulation as barcodes (a black line indicating the presence of the interaction at snapshot). (**D**) The binding energy (kJ/mol) between azm and VcCA. Hydrogen bonds are indicated in red dashed lines. H–arene interactions are indicated in yellow dashed lines. “A” indicates sidechain acceptor interactions, “a” indicates backbone acceptor interactions, “D” indicates side chain donor interactions, and “R” indicates H–arene interactions. Two interactions of the same type per residue (for example “A” for Gln102) indicates that multiple interactions of the specified type between ligand and residue are formed. The arrow indicates the sequence of snapshots of every 100 ps from 0 to 10 ns.

A 10 ns MD simulation was performed on the newly constructed VcCA homology model in complex with azm (Figure 3). The intermolecular interactions between the enzyme active site and azm were analyzed using the protein–ligand interaction fingerprint (PLIF) tool of MOE (v2019.01.02, Chemical Computing Group Inc., Montreal, Quebec, Canada). The main observed interactions during the simulation (represented as 100 snapshots; one snapshot per 100 ps) were with Gln102 (“A”: side chain acceptor interaction), Leu188 (“R”: H-Arene interaction), Thr189 (“D”: side chain donor interaction, “a”: backbone acceptor interaction), and Thr190 (“A”, “a”). The interactions of azm with the binding pocket of the VcCA model remained stable during the simulation, especially between the ligand’s sulfonamide group and the sidechain of Thr189 and Zn^2+^ (all snapshots). The hydrogen bond between azm and the sidechain of Thr190 was observed in 48% of the snapshots. The H–arene interaction between the ligand and the sidechain of Leu188 was observed in 37% of the snapshots. The hydrogen bond with the sidechain of Gln102 was only observed in 6% of the frames, including the first (0 ns) and the last (10 ns). The calculated average binding energy between azm and VcCA was 57.3 kJ/mol (Figure 3D).

### 2.2. Investigation of Compounds 4–24 as Possible Inhibitors of VcCA

**Figure 4:**
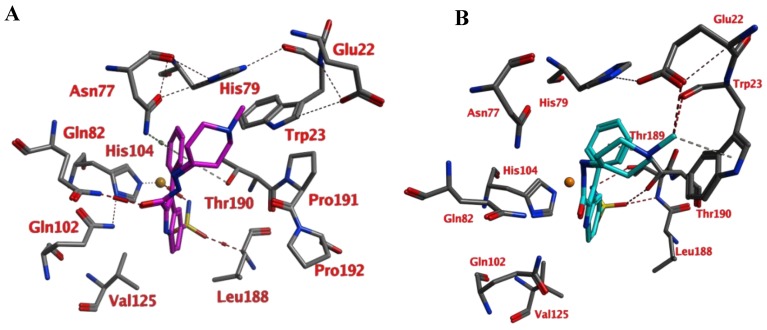

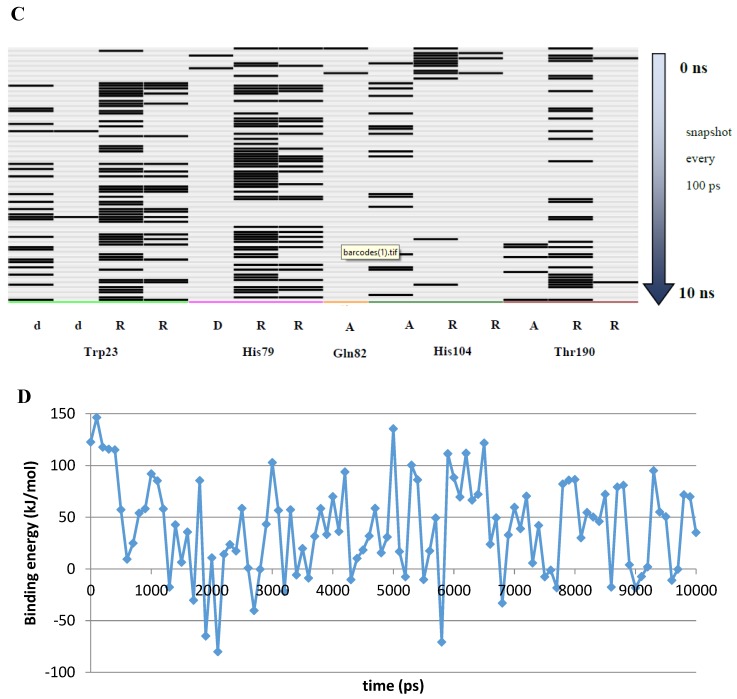
(**A**) The VcCA-23 structure obtained from docking (t = 0 ns). (**B**) The VcCA-23 model after a 10 ns MD simulation. (**C**) The protein–ligand interaction fingerprint (PLIF) showing the interaction of 23 with the VcCA binding pocket residues during the simulation as barcodes (a black line indicating the presence of the interaction at snapshot). (**D**) The binding energy (kJ/mol) between 23 and VcCA. Hydrogen bonds are indicated in red dashed lines. H–arene interactions are indicated in yellow dashed lines. “A” indicates sidechain acceptor interactions, “a” indicates backbone acceptor interactions, “D” indicates side chain donor interactions, and “R” indicates H–arene interactions. Two interactions of the same type per residue (for example “A” for Gln102) indicates that multiple interactions of the specified type between ligand and residue are formed. The arrow indicates the sequence of snapshots of every 100 ps.

Compounds of series 4–24 have been docked into the active site of the VcCA homology model. All ligand sulfonamides interacted with the active site Zn^2+^ ion as required by our docking protocol. As such, the common 3-phenyl-1*H*-indole-5-sulfonamide moiety of all compounds interacted in a similar way with the VcCA active site. The phenyl group may form hydrogen–arene interactions with the side chains of Asn77 and Thr190 (Figure 4A). Compound 23, which has the top-ranked docking score, shows a hydrogen bond with the sidechain of Gln82. The aliphatic ring forms hydrophobic interactions with the sidechains of Trp23 and His79. The linker between the aliphatic ring and the indole ring has an extended flat conformation. The indole NH group and the cationic N atom of the aliphatic ring are solvent exposed and most likely could form hydrogen bonds with water.

During a 10 ns MD simulation the interaction between the negatively charged nitrogen atom and the active site zinc ion remain stable even though no restraints between these two atoms were used (Figure 4). However, the hydrogen bond between the ligand carbonyl group and the Gln82 sidechain was not stable and lost early in the simulation. Instead, the sidechains of Trp23 and His79 adopt a different conformation that allows for a better accommodation of the ligands cationic aliphatic ring. This ring of the ligand forms H–arene interactions with mainly with the sidechains of Trp23 (50% of snapshots) and His79 sidechain (53% of snapshots). The distance between the cationic nitrogen atom to Trp23 and His79 is smaller than 5 Å for almost 90% of the time, thus, cation–π interactions are also possible. In addition, Glu22 moves closer to the cationic nitrogen atom during the simulation and 90% of the time the negatively charged oxygen atom of Glu22 is closer than 5.5Å to the cationic nitrogen atom. As such, a strong and long-ranged electrostatic binding interaction is present between 23 and the active site of VcCA.

The calculated average binding energy between 23 and VcCA was 40.2 kJ/mol (Figure 4D), this is lower compared to the binding energy observed for the VcCA-azm simulation. In contrast to the VcCA-azm MD simulation (Figure 3), a reorganization of the binding pocket was observed in the VcCA-23 simulation to allow for interactions with the ligand. As such, compound 23 was expected to bind to VcCA possibly with a higher *K*_i_ value.

As noted above, the docking studies suggest that the common 3-phenyl-1*H*-indole-5-sulfonamide moiety of all compounds interact in a similar way with the VcCA active site. In light of the MD results, it may be expected that the phenyl ring of this scaffold may form hydrogen–arene interactions with the side chain of Thr190. Smaller and neutral R groups may also allow for the formation of hydrogen bonds with Gln82. As such, compound 23 and possibly other compounds from this series are expected to show affinity for the VcCA active site.

### 2.3. Enzyme Inhibition Assays

**Figure 5:**
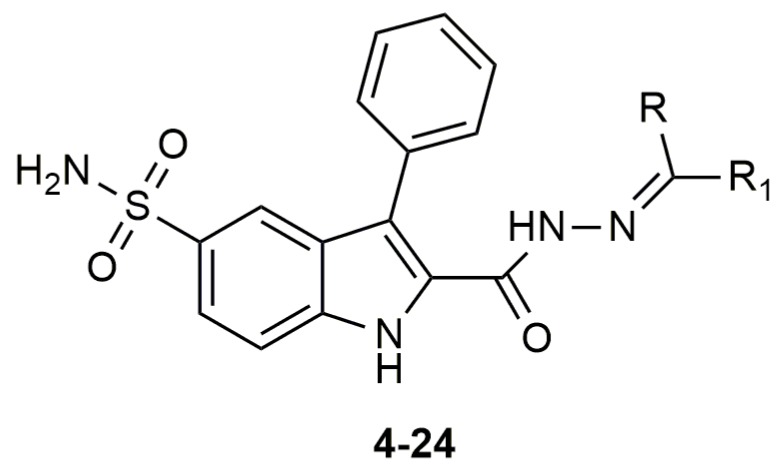
The molecular structure of compounds 4–24.

The compounds 4–24 and azm were tested in enzyme inhibition assays against VcCA and the data was compared to the previously obtained data of these compounds for the off-targets hCA I and II [34] (Figure 5, Table 1). A total of 19 of 21 compounds have *K*_I_ values lower than 100 nM against VcCA, including three compounds with a *K*_I_ values of less than 30 nM (i.e., compounds 11, 12, and 23). It is remarkable that for all compounds, the *K*_I_ values against hCA I and II are much higher, with the lowest *K*_I_ values being 3134.9 and 309 nM, respectively. Compared with VcCA, all compounds showed at least ≈10-fold and ≈4-fold selectivity over hCA I and hCA II.

Compound 23, carrying a piperidine ring with a methyl moiety formed strong interactions with the active site of the enzyme and was predicted as a potent inhibitor of VcCA in docking and molecular dynamic studies (Figure 4). In enzyme inhibition tests, compound 23 inhibited the VcCA enzyme with high selectivity and shows one of lowest inhibition values *K*_I_ **=** 25.2 nM.

Compound 12 with two methyl moieties at the tail part, has the best inhibitory activity (*K*_I_ = 22.8 nM) for VcCA and also has the best selectivity ratios for both off-targeted enzymes hCA I/II. Similarly compounds 10 and 11 that possessed small structures like methyl or hydrogen showed also good inhibition for VcCA (*K*_I_s = 46.6 and 27.1 nM, respectively). On the other hand, it is not beneficial to extend the aliphatic chain with ethyl or isobutyl for the VcCA inhibition, like compounds 13–15. 

In comparison, alicyclic or heteroaromatic substituted derivatives showed a moderate inhibition for VcCA, but cyclohexane with a *tert*-butyl (i.e., compound 22) or piperidine ring with a methyl group (i.e., compound 23 in Figure 4) was also well tolerated and led to increasing of the enzyme inhibitory activity.

Generally, all tested compounds showed good inhibitory activity towards VcCA in low nanomolar range and significant selectivity over hCA I and II. Compared to azm, new hydrazones had lower selectivity ratios for inhibiting VcCA against hCA I and II (36.7 and 1.76, respectively) (Table 1).

## 3. Materials and Methods

### 3.1. Construction of VcCA Homology Model

Homology models (100 models) of *Vibrio cholerae* α-CA enzyme (VcCA; Uniprot: A0A0X1L2C8) were constructed using the crystal structure of the α-CA from *Photobacterium profundum* (PpCA; pdb: 5hpj; 1.5 Å) including the azm coordinates from the hCA IX structure using the MOE software package (v2019.01.02, Chemical Computing Group Inc., Montreal, QC, Canada). The homology model with the highest contact score was selected and further optimized by steepest-descent energy minimization protocols (AMBER14:EHT force field). All heavy atoms of azm (acquired from hCA IX structure), the active site residues (all residues within 4.5 Å of azm), the zinc ion, the zinc-binding His residues, and the protein backbone were restrained, while the other protein atoms were unrestrained. Consequent minimizations with a stepwise release of the restraints was then performed. In the final step, the system was minimized without any restraints.

### 3.2. Preparation of Ligand Structures

Three-dimensional structures of the investigated ligands were prepared with MOE in low-energy conformations. The most prevalent protonation state of the ligands at pH 7 was calculated. The sulfonamide nitrogen atom was assigned a negative charge as this is the form in which this groups binds to the active site zinc ion. Subsequently, the ligands were energy minimized using the MMFF94x force field. 

### 3.3. Docking Studies into VcCA Homology Model

Docking studies were performed using the FlexX docking tool (v2.3.2; BioSolveIT GmbH, St. Augustin, Germany) within MOE. The binding pocket was described as all residues within 10 Å of azm (VcCA homology model). All ligands were docked 50 times and the highest scoring three poses were subjected to refinement calculations. To this end, the docked ligand and the binding pocket (defined as all residues within 6.5 Å of the docked ligand) were energy minimized and rescored using the GBVI/WSA force field [35]. 

### 3.4. Molecular Dynamics Simulations of VcCA-Ligand Complexes

The Yasara Structure software package (v18.8.9, YASARA Biosciences GmbH) was used for the molecular dynamics simulations with the PME method [36,37,38]. The selected docked poses (ligand-VcCA complexes) were first placed into the center of a cuboid box with periodic boundary conditions (minimal distance of 10 Å between protein and boundary). Afterwards, both water molecules (0.997 gr/mL; TIP3P) and counter ions (NaCl) were added to generate a solvated and neutral system. The system was energy minimized using a steepest-descent protocol (AMBER14) [39,40]. The system was energy minimization using steepest descent (100 cycles). Subsequently, the system was simulated for 10 ns at constant temperature (300 K, Berendsen, default values) and pressure (1 bar, Berendsen, default values), without any position restrains (production run). The only restraints applied were distance restraints to keep the zinc ion in the correct orientation towards nitrogen atoms of the zinc binding His residues (force: 100 N/m). The timestep was set to 2 × 1.25 fs and all bonds were constrained using the LINCS and SETTLE algorithms [41,42]. Snapshots were taken every 100 ps of the 10 ns production run. The binding energy (MM/PBSA) was calculated with Yasara Structure, while the RMSD values as well as the binding interactions (protein–ligand interaction fingerprint) were calculated with MOE.

### 3.5. Enzyme Inhibition Studies 

As previously reported, a stopped-flow instrument (SX.18 MV-R Applied Photophysics model) was used for assaying the CO_2_ hydration activity of various CA isozymes [43]. The 0.2 mm phenol red was used as indicator, working at the absorbance maximum of 557 nm with 10 mM Hepes (pH 7.4) as a buffer and 0.1 M NaClO4 (for constantly maintaining the ionic strength; this anion was not inhibitory in the used concentration) following the CA-catalyzed CO_2_ hydration reaction for a period of 5–10 s. Saturated CO_2_ solutions in water at 25 °C were used as substrates. Stock solutions of inhibitors were prepared at a concentration of 10 mM (in DMSO/water 1:1, v/v) and dilutions up to 0.01 nM were done with the assay buffer mentioned above. For allowing the complete formation of the enzyme-inhibitor adduct, the inhibitor and the enzyme were pre-incubated for 15 min. IC_50_ values were obtained from dose response curves working at seven different concentrations of the test compound (from 0.1 nM to 50 mM) by fitting the curves using PRISM (www.graphpad.com) and non-linear least squares methods, the obtained values representing the mean of at least three different determinations. The inhibition constants (*K*_I_) were derived from the IC_50_ values by using the Cheng–Prusoff equation as follows: *K*_I_ = IC_50_/(1+[S]/*K*_M_) where [S] represents the CO_2_ concentration at which the measurement was carried out, and *K*_M_ represents the concentration of the substrate at which the enzyme activity was at half maximal. All CA isoforms were recombinant ones obtained in-house as reported earlier [44,45,46,47,48].

## 4. Conclusions

In the present work, a novel series of 2-(hydrazinocarbonyl)-3-phenyl-1*H*-indole-5-sulfonamide incorporating hydrazone derivatives were investigated with docking and molecular dynamics studies for the purpose of discovering new potent inhibitors of α-class carbonic anhydrase of the pathogenic bacterium *Vibrio cholerae* (VcCA). Molecular modeling studies suggested that these compounds (especially compound 23) may bind to the active site of VcCA. Subsequently, all compounds were tested in VcCA enzyme inhibition assays. These molecules show strong inhibitory activity towards VcCA in low nanomolar range and display significant selectivity over hCA I/II. It is also remarkable that compound 23 which was suggested as a strong binder, inhibited the VcCA enzyme with one of the lowest *K*_I_ value, 25.2 nM and with high selectivity. 

## Figures and Tables

**Table 1:** Inhibition data against human (h) isoforms hCA I, II (cytosolic), and bacterial enzyme VcCA of derivatives 4–24 and azm by a stopped-flow CO_2_ hydrase assay.

Compound			*K*_I_ * (nM)			Selectivity Ratios	
	R	R_1_	hCA I	hCA II	VcCA	VcCA/hCA I	VcCA/hCA II
4	H	thiophen-2-yl	6667.5	>10,000	80.4	82.9	>124.3
5	H	5-Br-thiophen-2-yl	9408.7	>10,000	94.8	99.2	>105.4
6	H	1-CH_3_-pyrrol-2-yl	9572	>10,000	74.1	129.1	>134.9
7	H	pyridin-3-yl	8592.1	8906.7	74.2	115.7	120
8	H	pyridin-4-yl	3134.9	3487.4	72.1	43.4	48.3
9	H	indol-3-yl	>10,000	>10,000	60.5	>165.2	>165.2
10	H	H	7479.2	9369.7	46.6	160.4	201
11	H	CH_3_	4056.8	5866.8	27.1	149,6	216.4
12	CH_3_	CH_3_	9558.6	8498.4	22.8	419.2	372.7
13	CH_3_	C_2_H_5_	9619.6	8029.7	230.7	41.6	34.8
14	C_2_H_5_	C_2_H_5_	9246.3	6621.8	860.3	10.7	7.6
15	CH_3_	isobutyl	8652.3	6562.3	79.6	108.6	82.4
16	cyclopentyl		>10,000	5960.5	68	>147	87.6
17	cyclohexyl		>10,000	5867.9	81.4	>122.8	72
18	4-methylcyclohexyl		>10,000	8942.5	71.4	>140	125.2
19	4-ethylcyclohexyl		>10,000	1282.7	62.1	>161	20.6
20	4-propylcyclohexyl		>10,000	795.8	67.5	>148.1	11.7
21	4-phenylcyclohexyl		>10,000	830.7	52.1	>191.9	15.9
22	4-*ter*t-butylcyclohexyl		>10,000	3380.8	34.1	>293.2	99.1
23	1-methylpiperidin-4-yl		>10,000	309	25.2	>396.8	12.2
24	1-benzylpiperidin-4-yl		7384.7	372.4	92.4	79.9	4
azm	-		250	12.5	6.8	36.7	1.76

* Mean from three different assays, by a stopped flow technique (errors were in the range of ±5%–10% of the reported values). hCA I and II enzyme inhibition data obtained from previous studies [34].

## Abbreviations

CA	Carbonic anhydrases
VcCA	*Vibrio cholerae* carbonic anhydrase
hCA I/II/IX	Human carbonic anhydrases I/II/IX
PpCA	*Photobacterium profundum* carbonic anhydrase
azm	Acetazolamide
MD	Molecular dynamics
RMSD	Root-mean-square deviation
MOE	Molecular operating environment
*K*_I_	Inhibition constant
